# Recurrent Neural Network for Inertial Gait User Recognition in Smartphones

**DOI:** 10.3390/s19184054

**Published:** 2019-09-19

**Authors:** Pablo Fernandez-Lopez, Judith Liu-Jimenez, Kiyoshi Kiyokawa, Yang Wu, Raul Sanchez-Reillo

**Affiliations:** 1 University Group for ID Technologies (GUTI), University Carlos III of Madrid (UC3M), Av. de la Universidad 30, 28911 Leganes, Madrid, Spain; jliu@ing.uc3m.es (J.L.-J.); rsreillo@ing.uc3m.es (R.S.-R.); 2 Cybernetics and Reality Engineering Laboratory (CARE), Nara Institute of Science and Technology (NAIST), 8916-5 Takayama-cho, Ikoma, Nara 630-0192, Japan; kiyo@is.naist.jp; 3 International Collaborative Laboratory for Robotics Vision, NAIST, 8916-5 Takayama-cho, Ikoma, Nara 630-0192, Japan; yangwu@rsc.naist.jp

**Keywords:** Recurrent Neural Network, gait recognition, smartphone, pattern recognition, biometrics

## Abstract

In this article, a gait recognition algorithm is presented based on the information obtained from inertial sensors embedded in a smartphone, in particular, the accelerometers and gyroscopes typically embedded on them. The algorithm processes the signal by extracting gait cycles, which are then fed into a Recurrent Neural Network (RNN) to generate feature vectors. To optimize the accuracy of this algorithm, we apply a random grid hyperparameter selection process followed by a hand-tuning method to reach the final hyperparameter configuration. The different configurations are tested on a public database with 744 users and compared with other algorithms that were previously tested on the same database. After reaching the best-performing configuration for our algorithm, we obtain an equal error rate (EER) of 11.48% when training with only 20% of the users. Even better, when using 70% of the users for training, that value drops to 7.55%. The system manages to improve on state-of-the-art methods, but we believe the algorithm could reach a significantly better performance if it was trained with more visits per user. With a large enough database with several visits per user, the algorithm could improve substantially.

## 1. Introduction

Nowadays, it is more worrying to have our phones stolen than our wallets. In smartphones, we can find user information, bank accounts, pictures, personal contacts, addresses, etc. The nature and the amount of information stored have led to an increase of the value of our smartphones, making it imperative to increase their security. The first security methods implemented for smartphones were in the form of a personal identification number (PIN) or introducing a pattern on the screen [1]. Later on, biometrics were introduced in smartphone security as an improvement of both security and accessibility. This introduction came mainly in the form of fingerprint and facial recognition.

Biometrics uses the physical and behavioral traits of the user for recognition, eliminating the need to remember passwords or PINs. Moreover, presenting a biometric trait such as a fingerprint requires very little time or effort. These systems are so easy and secure that, in some cases, the implementations are considered secure enough to be used by bank accounts instead of a password or PIN. This security aspect did not arise mainly because biometrics are necessarily more secure than PINs but because users are more willing to use them.

Fingerprint and face recognition are based on a physical characteristic, but biometrics can also recognize how a user performs a specific activity. Behavioral biometrics—how the person behaves rather than a physical trait—are more difficult to imitate [2] and, depending on the implementation, can be invisible to the user. As of now, the major concern with behavioral biometric modalities is whether it would be able to be as accurate as physical biometric modalities [3], not only in terms of security, but also variability and spoofing. If they could get to the same level, they could work together with physical biometric modalities to increase security without yielding usability. There are several behavioral biometric modalities, such as voice recognition [4,5], handwritten signatures [6,7], key strokes [8,9], touch screen recognition [10,11], and gait recognition [12,13]. This article focuses on gait recognition, or the recognition of users as they walk. 

Gait is the manner of walking pattern of any given person. How we walk is unique as it depends on physical characteristics, the learning process and social environment. This makes gait a personal characteristic that can be used as a biometric trait [14]. This is a behavioral biometric modality since it requires the user to walk. Usually, requiring the user to do an action before recognition is contrary to usability; however, we usually walk with our phones either in a pocket or in a hand. Therefore, the user naturally performs the activity that is required for recognition even when the system is not in place. There are several ways of capturing this activity: the most commonly studied technologies in gait recognition are video recording [15,16], floor sensors [17,18] and inertial sensors attached to the user [19,20]. 

The best-known gait recognition technology is video gait recognition. Its implementation is meant for camera security scenarios, such as train stations or airports, when the image of the person under recognition is of low quality or is too unclear for face recognition. Nevertheless, it is also studied as a way to interoperate with face recognition [21]. Floor technology refers to the use of sensitive mats or surfaces that can measure the pressure and position of each step; this could be implemented as access systems to buildings or as a border control system. Finally, inertial sensor-based recognition requires the user to carry a device, commonly on the waist or upper thigh, to be recognized. Depending on the application, the system would use one of these technologies. Video and floor sensing can obtain more information from the user’s gait, since it can detect upper and lower movements; however, it requires the user to walk in a specific path, maintenance, connection, etc. On the other hand, inertial gait recognition can follow the user wherever it goes and does not require specific hardware (since virtually all smartphones have them embedded), but its information is more limited, and the approval of the user is required. As such, each system can be used in different scenarios. 

However, inertial gait recognition may prove to have more applications than meets the eye. For example, in the case of phone security, it is easy to understand its use. While the user walks, the smartphone would record the movement, and so by the time the user tries to access the phone, the system would already know whether to grant access or not. While it is true that the system requires the user to perform an activity, the user performs this activity anyway. However, the uses of this system do not have to be limited to smartphone access; with wireless communication, it could grant access to buildings or restricted areas, or be used for card payment security, etc. Furthermore, this system could be used not only as a security system but could also work with health care systems [22,23], adding an extra value to the access system.

This article presents a novel inertial gait recognition algorithm applied on a smartphone scenario. The algorithm works around a Neural Network (NN). Neural Networks have had proven results in other biometric modalities [24,25,26]. Related to this, Recurrent Neural Networks (RNNs) are used as a feature extraction algorithm. RNNs can determine different configurations, which is the main focus of this article: The possible configurations of RNNs and their results. The algorithms were compared to the state-of-the-art in inertial gait recognition. To do so, the algorithms were tested on a public database [27] which has been previously used as a benchmark due to its size of 744 users. 

This article will first present the related work and other algorithms in Section 2. Later, in Section 3, the database used is analyzed. In Section 4, the algorithm is presented. In Section 5, we establish the procedure to find the optimal configuration. In Section 6, we explain the training process of the NN algorithm. Section 7 gives the results of our algorithm and its configurations, and this is compared with the state-of-the-art methods. Finally, in Section 8, conclusions and possible future work are presented.

## 2. Related Work

As mentioned, gait recognition has several unique properties, such as operating with low-quality images, uncooperative recognition, ongoing recognition, and unobtrusiveness. When smartphone inertial sensors are used, not all these properties remain; nevertheless, some important ones that other biometric modalities may not have are still maintained, and new ones appear:

- User interaction: The implementation of a gait recognition system could run in the background. This means that, after setting it up, the user does not need to further interact with the system. Ideally, the system would recognize the user’s gait before even picking up the phone. For the user, seemingly, there would not be any control access.
- Hardware requirements: Biometric modalities such as a fingerprint need a specific sensor to capture the biometric trait. This sensor comes with more complications regarding to where to place it, the technology used, the size, etc. Even with face recognition, there is a need for a camera, sometimes even an infrared camera. Gait recognition uses the inertial sensors embedded in any smartphone. There is no need for any extra hardware, which means less production cost, less space required and a wider market.
- Spoof: In some articles, there has been proof that gait is difficult to mimic, even when the impostor is walking next to the captured user [28]. If not used as a standalone system, gait recognition can be used to detect spoof attacks and the liveness of the user.

Because of these features, inertial gait recognition has received a boost recently. The first implementation of inertial gait recognition was in 2005 by Ailisto et al. [29]; this was a simple implementation on an accelerometer sensor attached to the waist of the users. In 2010, the first implementation on smartphones was created by Derawi et al. [13]. The following years have been mainly dedicated to the improvement of algorithms in order to reduce error rates. Nevertheless, comparing results from algorithms that use their own databases can be complicated, as each can have different number of users, hardware configuration, conditions, etc., and Ngo et al. therefore created the Osaka University Database (OUDB) [27]. If we compare the OUDB with previous databases, as in Table 1, we can observe major differences between them. 

The OUDB has the greatest number of participants, while still maintaining an almost 50/50 proportion of genders. It utilizes both an accelerometer and gyroscope (which are commonly embedded in smartphones). Finally, the OUDB is open access, allowing for benchmarking. All these characteristics have allowed several algorithms to be tested on it, making comparison between algorithms more accurate. The most regarded algorithms for gait recognition have been studied in the OUDB. In addition, we have seen an evolution of algorithms from signal processing to machine learning (ML).

The algorithms of Gafurow et al. [30], Derawi et al. [31], Rong et al. [32], and Trung et al. [33] are the most regarded signal processing algorithms. They first detect the so-called gait cycles (GCs), which are the periodic movement of the gait, followed by a distance metric for comparison. Rong et al.and Gafurov et al. detect periods by measuring differences in the value of the acceleration, Derawi et al. uses a moving window and correlation, and Trung et al. applies Self-DTW (Dynamic Time Warping). All these methods simply divide the signal into its GCs. After extracting the GCs, the algorithms compare the cycles with the DTW distance, except for the case in Gafurov et al., which uses Euclidean distance. DTW was expected to work better in comparison due to its ability to compress and expand signals when comparing them.

Zhong et al. created the Gait Dynamic Image (GDI) as a new way to represent gait signals [34]. The GDI is a relation between the input values and its predecessors, meaning that the feature at time “t” is the comparison of that input with all previous inputs. This means that the gait signal is no longer a one-dimensional signal but rather an image, the width of which is the number of timestamps, and the height is the number of features used. The GDI is then processed by a Gaussian Mixture Model (GMM) and identity vectors, also known as i-vectors, are created. The use of the i-vector is a common practice for voice recognition and has shown good results.

Nguyen et al. [35] first represents the user’s walk in a matrix, then extracts features with a Convolutional Neural Network (CNN) and finally classifies it with a Support Vector Machine (SVM). This kind of NN works better when the input is two-dimensional, which is why the algorithm uses a projection of the signal in two dimensions. To do so, it segments the signal in portions by an empirical estimate of the GC length and projects the acceleration on different planes, transforming the signal into a matrix. By treating the matrix as an image, the algorithm then runs a CNN to extract the final features. Later on, those features are classified by the SVM.

Similarly, Delgado-Escaño et al. [36] also made use of a CNN. In this case, a walking signal is introduced raw into a CNN for feature extraction. This CNN outputs a feature vector of 744 components, one for each user. Finally, the feature vector is normalized. To obtain the comparison results, the feature vector is compared to all training samples by Euclidean distance; the distances are transformed to probability by normalizing by maximum distance and subtracting the result to one.

## 3. Database

The database, the OUDB, used to evaluate this algorithm was first presented in [27] by Ngo et al. The database is composed of two datasets: one on a leveled surface and the other on a sloped surface. Since there is still room for improvement in gait recognition on leveled surfaces, algorithms that have been evaluated on this database are always tested on the first dataset. 

**Figure 1:**
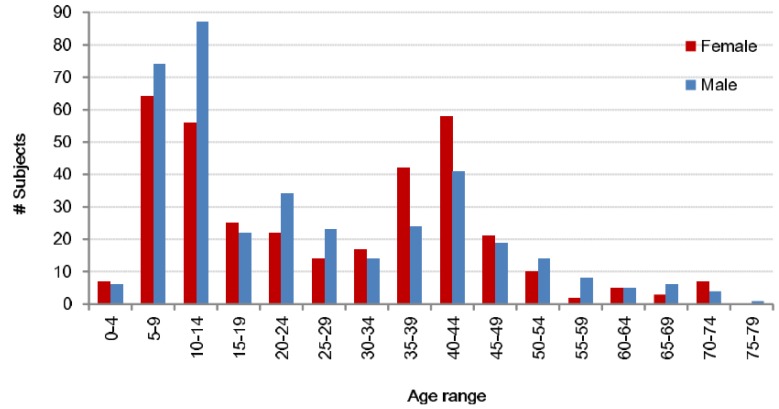
Gender and age distribution of the database.

The first dataset covers 744 users (389 males, 355 females), making it a realistic scenario. Also, the great variety of ages accounts for the influence of age (Figure 1). The users walked for 9 meters on a leveled surface. Each user walked two times in the same session. The second dataset is formed by a fraction of the previous users, 495 users (218 males, 277 females). In addition to the leveled surface walks from the previous dataset, these users walked up and down a slope of 8 degrees on separate occasions. These samples could be used to analyze the influence of walking on a sloped surface. The data of both datasets was obtained by an IMUZ sensor with a triaxial accelerometer and a triaxial gyroscope. The accelerometer had a dynamic range of ± 4 (g) and the gyroscope ± 500 (deg/s). All sensors had a sampling rate of 100 Hz and were recorded at the same time. The sensors were placed on the waist of the user via a belt.

The greatest feature of this database is the number of users, at 744 users, with an even proportion of male and female users, making it a realistic scenario. Also, the great variety of ages reduces the influence of age. Nevertheless, the number of samples is small for each subject. The shortness of the walking path makes it impossible to take full advantage of a biometric modality that can obtain data over long periods of time. In addition, since there are few samples per user, training any kind of ML algorithm to achieve good performance is not an easy task. Even so, the database has been used as a benchmark in recent years and is big enough to perform an evaluation of a gait recognition algorithm. 

When analyzing an NN, it is important to understand how the database is going to be divided. NNs, as with any ML algorithm, require a training phase. The standard approach that many ML algorithms follow is to train with the first sample of each user and then test with the second sample. This means that the samples used for training are also used for the enrolment of the users. A drawback of using this method is that there is no proof of scalability, meaning that if a new user was added to the database, there is no guarantee that the results would be maintained. This concern can always be raised regardless of how the database is split, but in this is case, it is more concerning since the training samples are simultaneously being used for enrolment. Thus, in this study, we intend to make use of the database in a different way by dividing the database by users rather than by samples. This will make comparison with previous algorithms more difficult, but it will also help to validate the scalability of the results.

## 4. General Algorithm Structure

**Figure 2:**
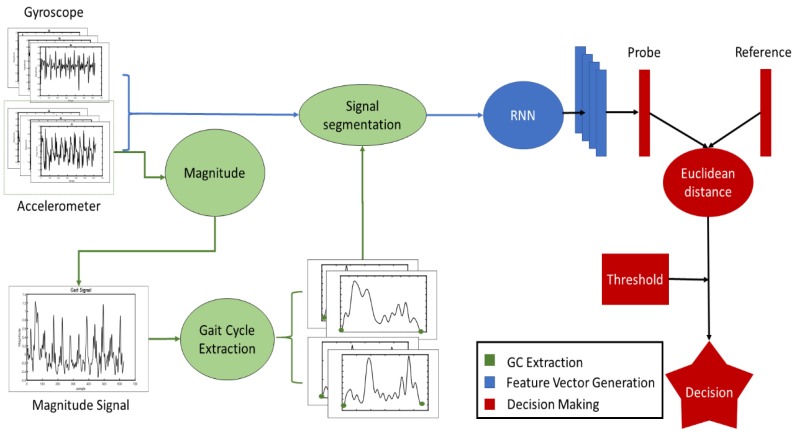
Algorithm schema. GC: gait cycle; RNN: Recurrent Neural Network.

To analyze the effectiveness of each RNN, a common structure is created. The proposed algorithm, as shown in Figure 2, has three differentiated processes: cycle extraction, feature vector generation, and comparison. As mentioned, the cycle extraction is a common step in many gait recognition algorithms in which the walking signal of the user is divided in sections to maximize the information and reduce the computational cost. Gait is a periodic movement—one step after the other and repeat—resulting in periodic gait signal. These periods are called gait cycles (GCs), the inertial values within two steps of the user. Therefore, the cycle extraction process refers to dividing the signal into these GCs. From the GCs, we generate a feature vector. The feature vector generation is performed by the corresponding RNN and transforms the gait cycle into a feature vector; this will be explained in detail in the next section. Finally, the comparison algorithm computes the distance between feature vectors to determine whether the user is recognized or not.

### 4.1. Gait Cycle Extraction

The signal has six different channels (A_X_, A_Y_, A_Z_, G_X_, G_Y_, G_Z_), where A_X_, A_Y_, and A_Z_ are the accelerometer values and G_X_, G_Y_, and G_Z_ denote the gyroscope values. All the channels have periodicity, but some are more difficult to differentiate than the others. This is due to not being in line with the movement of the leg. For example, we have found that the accelerometer has clearer periodicity than the gyroscope. The most discernable instance is the vertical axis of the accelerometer. However, since smartphones can be placed in any orientation, the vertical axis is not necessarily A_Y_ and can change every time the phone is used. This generates a problem, since the algorithm would first need to find the vertical axis. To simplify the problem, the proposed algorithm uses the magnitude signal (M) formed by the combination of the accelerometer values:

(1) $$M=\sqrt{A_{X}^{2}+A_{Y}^{2}+A_{Z}^{2}}.$$

The resulting signal is centered by subtracting the mean value of the signal. The signal has a well-defined periodicity, and since it does not have a time shift with respect to the original signals, it can be used to determine the start point of cycles. It is important to note that this signal will help define the points at which the periodicity starts, but this signal does not go through feature extraction. 

**Figure 3:**
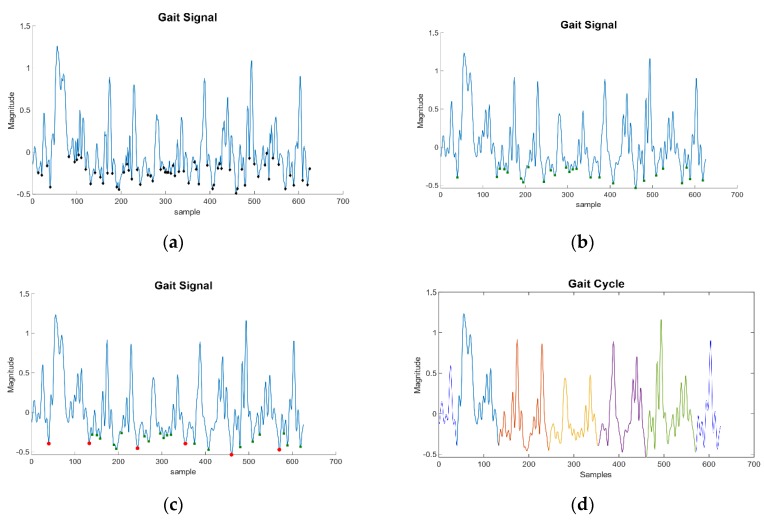
Cycle extraction process. (**a**) local minima, (**b**) magnitude remaining, (**c**) delimiting starting cycles, (**d**) final cycles (differentiated by colors).

Once M is obtained, the algorithm can look for the starting points of a cycle. In this case, the algorithm looks for the distinctive minima that correspond to the user stepping their foot on the ground. This point has been selected since it has a clear distinction compared to other possible starting points. To find these points, the algorithm makes a discard method:

- First, the algorithm looks for all negative minimum peaks, as shown in Figure 3a. In this step, several values are marked, most of which are simply characteristic points of the walking signal. Within these points are the starting points of a GC, but many more are in the list that need to be discarded.
- The mean of the values in the previous step is computed. All points above that value are removed from the candidate list. At this stage, the points usually belong to the beginning of each step, as shown in Figure 3b. However, a gait cycle is composed of two steps, and so there are candidates that do not limit a GC.
- Lastly, the time difference between points will determine the gait cycles, as shown in Figure 3c,d. Points that are considered to be too close are removed from the candidate list by the algorithm. By doing so, the algorithm manages to find the final points that determine the beginning of new GCs.

The timestamps of the starting GCs of M are the same as those in the original six-axis signal. As such, we can divide the six-axis signal into the gait cycles and use those as inputs of the feature extractor. With this, the session of a user can be defined as

(2) $$S_{i}^{j}=\left\{GC_{1},GC_{2},\ldots ,GC_{N}\right\},$$

(3) $$GC_{n}=\left\{A_{X},A_{Y},A_{Z},G_{X},G_{Y},G_{Z}\right\},$$

where $S_{i}^{j}$ is the jth session of the ith user and $GC_{n}$ is the gait cycle n of the session.

It is important to note that not all GCs are of the same length. Depending on the speed of the user, GCs can become shorter. GCs can have a size variation from 90–120 inputs. By accommodating this disparity in size, we ensure that gait cycles have all the data of that cycle.

### 4.2. Feature Vector Generation

After the signal has been segmented, the algorithm will extract the feature vectors of the GCs. To do so, the GCs will go under one of the RNNs of long-short-term memory cells (LSTM) that are under study. RNNs have been widely used to process data sequences in several fields, from music generation to speaker recognition. In this case, the RNN will create a feature vector of the GC to make it easier to discern users.

Although the exact hyperparameters change, all the algorithms follow the same structure. The algorithm starts with an RNN on LSTM cells, and in some cases, several RNNs would be placed in a pipeline schema. After the last RNN, there should be a fully connected (FC) layer; in some cases, there might not be a FC layer, while in other cases there are several FC layers. The number of filters and neurons may vary in each configuration.

As mentioned, the GCs are not all of the same length; for this reason, dynamic RNNs are used. The maximum input length is 120 inputs; any GC of shorter length would be filled to this size by padding with zeros. Each input is in the form of six dimensions (A_X_, A_Y_, A_Z_, G_X_, G_Y_, G_Z_). For simplicity, GCs are represented as 120 × 6 inputs, even when the GC can be shorter. The output of the full NN outputs a single vector, whose size will vary depending on the NN. Once all cycles of a visit are processed, the visit would have N number of vectors, each for a GC. The final feature representation of the visit would be the mean value of all feature vectors:

(4) $$S_{i}^{j}=mean\left(\left\{v_{1},v_{2},\ldots ,v_{N}\right\}\right),$$

where $v_{i}$ is the output vector from the RNN and $S_{i}^{j}$ the final vector of the session, resulting in the vector of mean values. The comparison between these final vectors is done using Euclidean distance. Unlike other methods, this algorithm does not need DTW for it is comparison of fixed feature vectors that are not time dependent.

## 5. Recurrent Neural Networks

Finding the right NN for a specific algorithm can be complex. For a new algorithm, there is no reference for the number of layers, number of neurons, filters, fully connected layers, etc. There are different approaches to find the optimal hyperparameters. In the case of this algorithm, the random grid followed by hand-tuning has been chosen.

The random grid is an iterative process in which hyperparameters are established between a minimum and maximum value. Once the limits of each hyperparameter are selected, values for each of them are selected at random 25 times. These configurations are tested on a train/test ratio of 60%/40% of users (meaning that the sessions of 447 users are used as training and the other 297 as testing). Results of the different samples are compared, and the one with better performance is selected as a new center point for a new random grid with a smaller size. This process is repeated until a small, manageable grid is obtained. 

At that point, hyperparameters are hand-tuned, which means modifying a single parameter in each iteration, looking for the optimal result. In this case, the results tend to be similar between configurations. To obtain a clearer view of the results of a configuration, the configurations are tested in different train/test ratio (from 10%/90% to 90%/10%) of the database and tested 5 times for each train/test ratio. The configuration is evaluated by their mean values and the progression as more training data is added.

The used random grid with values is shown in Table 2, in which the hyperparameters are those which are modified in each iteration to fine-tune it. These values are intentionally wide, as the intention at this point is not to find the ideal configuration, but rather the area in which it could be found. After 25 random selections, new limits are established, and the process is repeated. From the resulting 25 selections, the one with lowest error was selected for hand-tuning. The hand-tuning process modifies a single hyperparameter of the configurations and obtains results. Once no progression is detected, we have reached the final configuration of our algorithm.

## 6. Training the Neural Networks

The training process of the RNN is mainly determined by its loss function. Many loss functions are characterized by classification methods, meaning that the loss value is determined by the probability of the output belonging to a specific class. In our case, however, there are no classes. We could use each user as a different class, but since the users in the training phase are different from the testing phase, the classes would lose meaning. The loss function that is required in this case needs to make a feature space in which samples of the same user are close to each other while being separate from other users.

One of the functions that fulfills this condition is the triplet loss function [37,38]. This function works in triplets of units. This means that the algorithm needs three inputs: an anchor, a positive probe and a negative probe. The algorithm calculates its loss as the distance between the anchor and the positive probe and negative probe. The objective is to make the distance between the anchor and positive probe small relative to the distance between the anchor and negative probe. This would mean that the features of the same user stay in a small, closed space which is distant from the space of any other user. The mathematical equation is

(5) $$Loss=\sum_{i=1}^{N}\left[\|f_{i}^{a}-f_{i}^{p}{\|}_{2}^{2}-\|f_{i}^{a}-f_{i}^{n}{\|}_{2}^{2}+\alpha \right],$$

where $f_{i}^{a}$ is the anchor at iteration i; $f_{i}^{p}$ is the positive probe at iteration i; $f_{i}^{n}$ is the negative probe at iteration i; and α is the margin.

As can be seen, the first term calculates the distance between the anchor and positive probe, the second one is the distance between the anchor and negative probe, and finally there is the margin α. The first factor determines the difference between the anchor and the positive probe, and the larger the distance, the bigger the loss. The second factor determines the distance between the anchor and the negative probe; in this case, a larger distance results in a smaller loss. The margin is the only hyperparameter for this cost function; thus, we establish how great of an error margin we want to give to the algorithm. In our particular case, α=100. 

To apply the triplet loss function, the algorithm would need enough triplets (anchor, positive and negative); however, as mentioned, the OUDB has a major disadvantage in that the number of samples per user is small. To solve this problem, the algorithm was trained by individual GCs rather than entire walking signals. Thus, for each session, the algorithm could train with several GCs; usually 4–5. This multiplies the number of samples considerably. In addition to the triplet loss function, there must be a mechanism to avoid overfitting the NNs. In the case of the study, the dropout method is used. The dropout method “blocks” certain neurons during the training phases. By doing this, the NN is forced to distribute information among different neurons and features, rather than depending on a subset of the information. We implemented an 80% chance of keeping neurons. This means that, in each training step, an individual neuron has a 20% chance of being unused. 

Finally, in this study, we divide the database by users rather than by samples. This means that when using 10% of the database, we refer to using all the visits of 74 users (and 670 users for testing). Another advantage of using this division is that the algorithm is less likely to overfit the trained users. If we used the same users for training and testing, it would be difficult to ensure the scalability of the algorithm.

## 7. Results

When a biometric algorithm receives an access attempt, the algorithm makes the required comparison and obtains a value. If this value is below the threshold, in the case of distance-based algorithms such as ours, the attempt is recognized; if not, the attempt is rejected. When using a restricting threshold value, more attempts are rejected, creating a high false non-match rate (FNMR): rejecting attempts that should have not been rejected. If the threshold is less restrictive, it could create a big false matched rate (FMR): attempts that should have been rejected are actually not rejected. For a specific threshold value, the FMR and FNMR have the same percentage of error. We call this percentage the equal error rate (EER). EER has been used to compare algorithms since it is easy to determine and shows a good approximation for how the algorithm will work. A lower EER can indicate a better-performing algorithm. We will use this value throughout the results in order to compare algorithms.

Another point of consideration here is the distribution training/testing of the database. When using any ML algorithm, there is a discussion regarding the percentage of the database to use for training and testing. The more training samples, the more accurate it can be; however, the more training samples, the less statistical significance the testing results will have, since there are fewer samples for testing. Furthermore, overfitting is also a concern; when using the same database for training and testing, it might happen that the algorithm has only learnt how to solve this database but cannot generalize. Therefore, to obtain a more detailed understanding of the algorithm, different ratios of training/testing need to be studied.

The results section is divided into three:

- In “Random Grid Results”, we will first analyze the EER of the different algorithm hyperparameter configurations. In this section, we will only focus on EER values, since the obtained configuration is still not the final one. Furthermore, the results will be compared on a 60% train/40% test configuration.
- Hand-tuning results first shows the EER values of the configurations obtained from hand-modified hyperparameters. In this section, results for all train/test configurations are represented. These results are the average of five different training instances.
- In the benchmark section, we compare the results obtained in the final algorithm with other state-of-the-art results. We use the EER published by previous algorithms as well as ours. We use these results to try to determine how gait recognition on smartphones is evolving as a whole.

### 7.1. Random Grid Results

To find the optimal NN configuration, we first take a random grid approximation, as explained in Section 5. In each process, we configure the algorithm as one of the possible configurations within the values of Table 2; we do this randomly, as an intensive search is computationally costly. The configuration is trained with 60% of the users and tested on the other 40%. The EER obtained in the test is then plotted in Table 3.

After testing 25 random configurations, it can be seen that the NNs with fewer layers and fewer neurons per layer have the lowest EER values. Overall, it can be observed that the central configurations tend to perform better. To move forward in the search for an optimal configuration, we now limit the range of possible configurations. From Table 3, we can determine that three layers of RNN do not have the best performance; therefore, we would limit the search to two layers. Moreover, focusing on the number of FC layers, there is no clear relationship between the number of layers and EER values; therefore, we continue with the same limitations of FC. In the case of both filter and vectors, it can be seen that a low number of filters or vector dimensions (under 16) seems to have a negative impact and high values seem to have the same effect, but since this is not very clear, we prefer to maintain the limitations. We configure new limit values and resample 25 different random configurations. 

From the 25 new values, we look for the best-performing configuration to use as a starting point for hand-tuning. The configuration of RNNL: 1, FCL: 2, number of filters: 32, vector size: 64 had a significant lower value compared to other configurations with a 7.54% EER. Although this is a good result, there could still be improvement. We must also consider that different trainings could lead to different results due to the convergence of the training. Lastly, we would also like to see the progress of the algorithm when using different train/test distributions other than 60/40. For these reasons, we now hand-tune the algorithm.

### 7.2. Hand-Tuning Results

To analyze the algorithm more exhaustively, two measures were taken: First, the algorithm was tested in different proportions of users for training (from 10% to 90% training); secondly, each proportion was tested five times. These two elements made sure that, even with the algorithm’s uncertainty, we obtained consistent results as well as understanding the influence of the amount of data used for training.

**Figure 4:**
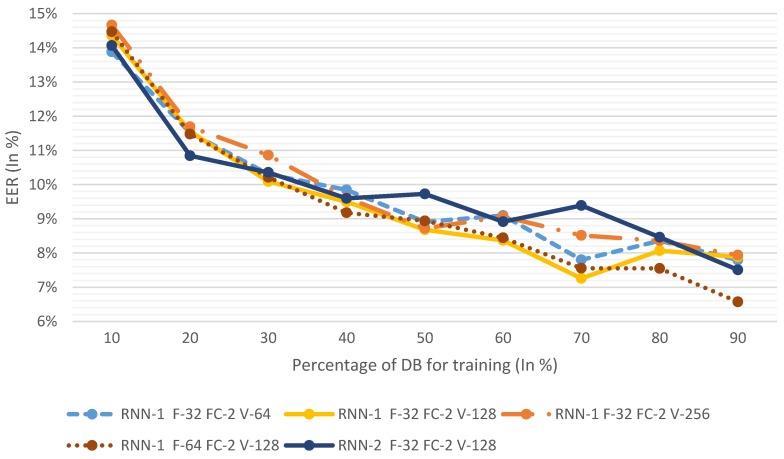
Hand-tuning equal error rate (EER). DB: database.

In Figure 4, the results of all hand-tuned systems are represented. To explain why we decided to use these algorithms, and what the results represent, starting with RNN1_F32_FC2_V64—which is the one obtained in the random grid—we can observe what we could expect from the ML algorithm, namely that the bigger the number of samples for training, the lower the errors. Furthermore, we see that the minimum error is actually obtained at 70% of users for training; this could mean that at 80% and 90%, the number of testing users might be too low (147 and 73, respectively). In any case, the progression of the algorithm behaves normally. 

After testing the algorithm and obtaining the results, we now increase the complexity of the algorithm by increasing the vector output size to 128, making the configuration RNN1_F32_FC2_V128. As seen in the figure, this configuration clearly has a reduced EER as it gets lower error rates on the highest number (4 of 9) of train/test distributions. Since there is an improvement by increasing the vector size, we increase it once more, obtaining the configuration RNN1_F32_FC2_V256. In this case, EER increased by a significant margin for all distributions, which shows that a vector size of 128 performs better. 

Since the vector size has reached its limits, we now increase the number of filters with the configuration RNN1_F64_FC2_V128. In this case, the results at first glance are not so clear regarding whether there was an improvement or not (Figure 4, yellow solid line and brown dotted line). In small training samples (< 50%), RNN1_F32_FC2_V128 has lower EERs; however, at 50–70%, the results of both configurations are very similar, and at 80–90%, RNN1_F64_FC2_V128 shows lower EER values. Taking it into account that this is a small database for an NN, and that the difference in EER seem to favor RNN1_F64_FC2_V128 for further scalability, this configuration is considered to perform better. 

As a counter to increasing the width of the NN (the number of filters), we now increase its depth by adding a layer of RNN with the configuration RNN2_F32_FC2_V128. The results show that the increase of depth does not improve the EER. It would seem that the best-performing configuration would be RNN1_F64_FC2_V128. To fully understand the results of the algorithm proposed in this paper, we study its results in depth in Table 4. 

We now compare our algorithm with other state-of-the-art methods; several algorithms have been tested in this database, and we compare our algorithm with the ones explained previously.

### 7.3. Benchmark

With these observations, we can compare the final algorithm with the ones previously mentioned; the comparison is shown in Table 5. The differences of the training and testing distribution in each algorithm are worth noting. We have established the number of users with the information presented in each of the articles. Those algorithms that do not use training in their algorithm are indicated with “-“. The differences in the number of users used for testing are shown as they may influence the EER of each study. For example, Nguyen et al. used a sample of the users in order to train their CNN algorithm and reported different results for different ratios training/testing, as in our study. In the case of Zhong et al., they trained with one visit of each user and then tested with the other visit. The results obtained by previous algorithms were obtained by the corresponding articles in their references.

Thanks to the OUDB, we can observe the progression of gait recognition over time. As time has passed, gait recognition has gone from a 20.20% EER to only 1.14%. It can be seen that the performance of gait recognition algorithms is improving with time. This would mean that gait recognition is not in a stagnant position and could improve even more in the following years.

With the classical algorithms, we can observe that EERs were between the 20% and 14% marks. This is understandable, as the amounts of information available at those stages were small; each study would use their own dedicated DB with less than 50 users. However, with the creation of the OUDB, researchers have managed to study algorithms in a wider range. This is made clearer with the introduction of ML. The first to make use of them were Zhong et al., who created an algorithm with a 5.60% EER. This improvement was increased by Delgado-Escaño et al., who attained a 1.14% EER. Nevertheless, these results were obtained in a closed set environment, using one visit for training and enrolment and another for testing. Our use of the database differs from this since we divide the database by users rather than visits.

Our results would more accurately compete with those of Nguyen et al., as with a similar portion of users, we obtain similar performances. It can be said that both algorithms performed similarly since EERs are similar, with the lowest values those from Nguyen et al.; however, a comparison with Nguyen’s algorithm is complicated since they did not test with larger proportions of training, making it difficult to estimate their scalability. For future reference, we include the EER of 70% training as it would seem that more data did not improve performance, making the best performance of our algorithm 7.55% EER. These EERs, although not the lowest on the table, represent a better view of how the problem of gait recognition could be improved in future studies.

## 8. Conclusions

This article has presented a new approach to gait recognition system for smartphones by introducing the RNN. The system records inertial data from the user, generating six gait signals, and then it fragments those signals into gait cycles to later transform them into feature vectors by means of an RNN. These feature vectors are then combined by obtaining the mean cycle, which is used as a template for the users. This algorithm could be applied in a variety of scenarios, such as smartphone control access or building control access, whether together with another biometric algorithm or as a standalone. This would be an invisible system for the user, since it does not require a direct interaction. 

Under that common structure, the RNN was developed and studied. To find the optimal configurations of the NN, a random grid followed by a hand-tuning approach was used. Firstly, we parametrized several hyperparameters and established boundaries for the values of each hyperparameter. From those boundaries, random values where chosen and tested. From those tests, we reduced the boundaries and chose a new, random selection. From then onwards, each parameter was hand-tuned to find better performances, arriving at the final algorithm. Not only was the optimal configuration shown, but also the results of less optimal configurations, making it possible for future research to discard those approaches.

The algorithm was tested in a benchmark and compared to seven different algorithms. The results obtained were competitive with the state-of-the-art methods, reaching an EER as low as 7.55%. More importantly, this study performed the division of the database by user rather than by visit, aiming for a more comprehensive view of the scalability of the problem. We have studied how the algorithm lowers its error rate by adding more users, creating a tendency which suggests that if more users were to be added to the database, the algorithm would perform even better.

Even so, an EER of 7.55% does not compete with other biometric modalities used in smartphone recognition such as fingerprint or facial recognition. Nevertheless, gait recognition is still a young biometric modality, and it will probably work along other biometric modalities in the near future. 

ML algorithms have shown better results in terms of biometric modality. It is safe to say that future studies on gait recognition should use NNs or some other short of ML in order to improve on the state-of-the-art methods. One of the main issues with these algorithms is the amount of data needed for training. Algorithms such as ours that use NN require large databases. For now, the largest database is the one used in this article, with 744 users; the number of users might be sufficient, but the number of visits is not high enough. With this number of users, we can clearly study the intervariability of the sample, but not the intravariability, since we do not have several samples per user. For gait recognition to move forward, more databases with more samples per user are needed.

Overall, gait recognition on smartphones is starting to achieve competitive results which can be used alone or in combination with other biometrics.

## Figures and Tables

**Table 1:** Databases used in the previous articles.

Name	Users (Male/Female)	Visits Per User	Sensors	Dynamic Range	Frequency (Hz)	Path Size (m)	Sensor Position
Ailisto et al. [29]	19/17	2	Accelerometer	-	256	20	Waist Back
Gafurov et al. [30]	30	4	Accelerometer	-	100	20	Ankle
Derawi et al. [31]	60	12	Accelerometer	±6g	100	20	Waist left
Rong et al. [32]	11/10	5	Accelerometer	-	250	30	Waist Back
Trung et al. [33]	25/7	5	Accelerometer Gyroscope	-	100	2 min	Back
Zhong et al. [34]	20	2	Accelerometer	-	-	-	-
Ngo et al. [27]	389/355	2	Accelerometer Gyroscope	±4 g ±500 deg/s	100	9	Waist Back

**Table 2:** Hyperparameter random grid.

Variable	Values
Number of RNN layers	[1,3]
Number of Fully Connected Layers	[0,3]
Number of filters	2^[1,10]^
Feature Vector size	2^[1,10]^

**Table 3:** Equal error rate (EER) results from the random grid approach (%). V.S. = vector size, N.F. = number of filters, FC = fully connected.

	RNN Layers	1	1	1	1	2	2	2	2	3	3	3	3
	FC Layers	0	1	2	3	0	1	2	3	0	1	2	3
V.S.	N.F.												
*2*	64		20.4										
*4*	8						14.0						
*4*	256	50.9		48.0									
*8*	8						25.9		12.7				12.7
*16*	8					13.2			12.4				
*16*	16				10.3			10.0					
*16*	512		10.3										
*32*	2	23.6											
*32*	4			13.4									
*32*	128												26.3
*64*	2									24.0			
*64*	8								13.7				
*128*	128									50.0			
*256*	256	8.9											
*512*	4										26.3		
*512*	256											46.3	
*1024*	2	14.4											
*1024*	8						11.3						
*1024*	64											49.4	
*1024*	128							48.3					

**Table 4:** Detailed EERs of the final algorithm, Rnn-1 F-64 FC-2.

PERCENTAGE OF DB FOR TRAINING	10%	20%	30%	40%	50%	60%	70%	80%	90%
1	17.50	11.59	10.43	8.93	9.40	7.86	7.62	6.88	6.66
2	13.82	11.69	9.85	9.38	8.78	8.72	8.33	8.08	6.64
3	12.88	11.16	10.12	9.65	8.82	7.96	7.94	6.69	6.36
4	14.70	12.28	9.74	9.88	9.38	9.32	7.17	8.72	6.82
5	13.44	10.67	10.93	8.05	8.31	8.37	6.71	7.37	6.40
AVERAGE	14.47	11.48	10.21	9.18	8.94	8.44	7.55	7.55	6.57
MINIMUM	12.88	10.67	9.74	8.05	8.31	7.86	6.71	6.69	6.36
STD	0.13	0.01	0.01	0.02	0.01	0.01	0.02	0.03	0.00

**Table 5:** EERs of benchmark methods.

	Training	Testing	Results (EER)
**Trung et al.** [33]	-	744	20.20%
**Gafurov et al.** [30]	-	744	15.80%
**Rong et al.** [32]	-	744	14.30%
**Derawi et al.** [31]	-	744	14.30%
**Zhong et al.** [34]	744 ^1^	744 ^1^	5.60%
**Delgado-Escaño et al.** [36]	744 ^1^	744 ^1^	1.14%
**Nguyen et al.** [35]	60/140	744	10.64%/10.43%
**RNN-1 F-64 FC-2 V-128 (PROPOSED METHOD)**	148/520	596/224	11.48%/7.55%

^1^ One visit for training and enrolment, one visit for testing.
